# Modification of AgNP-Decorated PET: A Promising Strategy for Preparation of AgNP-Filled Nuclear Pores in Polymer Membranes

**DOI:** 10.3390/ijms25020712

**Published:** 2024-01-05

**Authors:** Jakub Siegel, Daniel Grossberger, Jana Pryjmaková, Miroslav Šlouf, Petr Malinský, Giovanni Ceccio, Jiří Vacík

**Affiliations:** 1 Department of Solid State Engineering, University of Chemistry and Technology Prague, 166 28 Prague, Czech Republic; dan.grossberger@seznam.cz (D.G.); jana.pryjmakova@vscht.cz (J.P.); 2 Institute of Macromolecular Chemistry, Academy of Sciences of the Czech Republic, Heyrovského nám. 2, 162 06 Prague, Czech Republic; slouf@imc.cas.cz; 3 Department of Physics, Faculty of Science, University of Jan Evangelista Purkyně in Ústí nad Labem, 400 03 Usti nad Labem, Czech Republic; malinsky@ujf.cas.cz; 4 Department of Neutron Physics, Nuclear Physics Institute of the Czech Academy of Sciences, 250 68 Husinec, Czech Republic; ceccio@ujf.cas.cz (G.C.); vacik@ujf.cas.cz (J.V.)

**Keywords:** polymer, silver nanoparticle, laser, immobilisation, physical modification, ion-track membrane

## Abstract

Polymer-based membranes represent an irreplaceable group of materials that can be applied in a wide range of key industrial areas, from packaging to high-end technologies. Increased selectivity to transport properties or the possibility of controlling membrane permeability by external stimuli represents a key issue in current material research. In this work, we present an unconventional approach with the introduction of silver nanoparticles (AgNPs) into membrane pores, by immobilising them onto the surface of polyethyleneterephthalate (PET) foil with subsequent physical modification by means of laser and plasma radiation prior to membrane preparation. Our results showed that the surface characteristics of AgNP-decorated PET (surface morphology, AgNP content, and depth profile) affected the distribution and concentration of AgNPs in subsequent ion-track membranes. We believe that the presented approach affecting the redistribution of AgNPs in the polymer volume may open up new possibilities for the preparation of metal nanoparticle-filled polymeric membranes. The presence of AgNPs on the pore walls can facilitate the grafting of stimuli-responsive molecules onto these active sites and may contribute to the development of intelligent membranes with controllable transport properties.

## 1. Introduction

Polymer membranes are thin sheets of material typically composed of synthetic polymers such as polyacrylonitrile (PAN), polyimide (PI), polycarbonate (PC), polyethylene (PE), polypropylene (PP), polytetrafluoroethylene (PTFE), and polyethyleneterephthalate (PET) [1]. For specific applications, natural-based polymers such as cellulose acetate (CA), chitosan, and polysaccharides [2] may be used as they are or in the form of bionanocomposites doped with foreign species such as inorganic or organic nanoparticles [3]. Polymeric membranes are employed in numerous applications involving transport processes, more specifically in the barrier properties of packaging films [4]; membrane separation processes for liquid and gaseous mixtures [5]; water purification processes, including reverse osmosis and nano-, ultra-, and microfiltration [6,7]; and dialysis [8]. Emerging applications employ polymeric membranes in fuel cell and lithium battery applications [9].

Not only the polymers themselves, but also their composites, are gaining attention in membrane technologies. These types of membranes are known as mixed matrix membranes (MMMs). MMMs are an innovative class of materials revolutionising the landscape of membrane technology, particularly in separation processes. These membranes amalgamate two distinct components, a polymeric matrix and dispersed filler materials such as nanoparticles [10,11], as they are or attach to carriers supporting pore formation such as zeolites [12,13] or other porous materials [14]. This unique combination harnesses the advantages of both constituents, resulting in enhanced separation efficiency, mechanical strength, and versatility, making MMMs a promising solution across various industries. The basic characteristics of polymer membranes, such as pore density, size, and shape, are also affected [15]. These are the basic parameters that determine the specific application and selectivity of the membrane.

Furthermore, the development of membranes is constantly evolving, with ongoing research focused on optimising filler materials, improving compatibility between the matrix and fillers, and improving fabrication techniques to achieve higher performance and scalability. There are several methods used in the preparation of MMMs, each with its advantages and suitability for different types of polymer, filler, and intended applications. Some common methods include solution casting [16,17], in situ polymerisation [18], electrospinning [19,20], and layer-by-layer assembly [21,22]. Currently, a quite challenging task is to enhance selectivity and enable activation of membrane capabilities by external stimuli [23]. To meet these criteria, membrane pores can be filled with pH-, temperature-, or electromagnetic radiation-sensitive molecules, which upon change of conformation may affect the transport properties. Diverse approaches have been employed to anchor the active molecule to the pore wall, typically based on covalent attachment mediated by physical modification such as chemical pre-treatment or UV or plasma irradiation [24]. However, they often do not provide satisfactory results because of the complicated shape of the pores and inaccessibility that causes weak bonding and gradual release of pore fillers.

In this work, we present a novel approach to introduce active sites into the pore walls of ion-track PET membranes in the form of silver nanoparticles (AgNPs). The presented technology is based on the combination of two consecutive processes prior to nuclear pore formation: (i) firm immobilisation of AgNPs, homogeneously spread over the polymer surface, and (ii) surface modification of AgNP-immobilised PET, which affects the surface morphology of the polymer and causes redistribution of Ag particles in the thin surface region of the vertical polymer profile. The quality of the polymer surface is crucial during the process of pore formation by swift ion irradiation and subsequent etching of latent ion tracks. The distribution of AgNPs in the thin surface layer of the polymer fundamentally affects the concentration and distribution of nanoparticles in the pores of the ion-track polymeric membrane. Here, we primarily focus on the modification of planar AgNP-doped PET foil prior to the pore formation process, which appears to be a promising alternative to produce AgNP-filled pores in PET membranes with etched ion tracks. Optimisation of the modification parameters and nuclear pore formation will be studied in detail in our further study.

## 2. Results and Discussion

**Figure 1:**
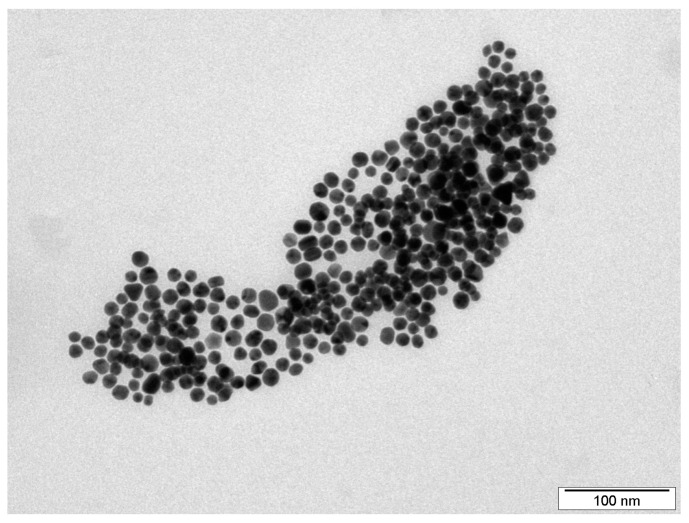
TEM image of electrochemically synthesised silver nanoparticle colloid subsequently used in the laser-assisted immobilisation process.

The shape and size of the synthesised silver nanoparticles were characterised by TEM. Figure 1 shows that the AgNPs were mostly spherical, with an average size between 20 and 25 nm. Such particle parameters are essential for subsequent immobilisation by LIFT technology using polarised light from a KrF excimer laser [25].

**Figure 2:**
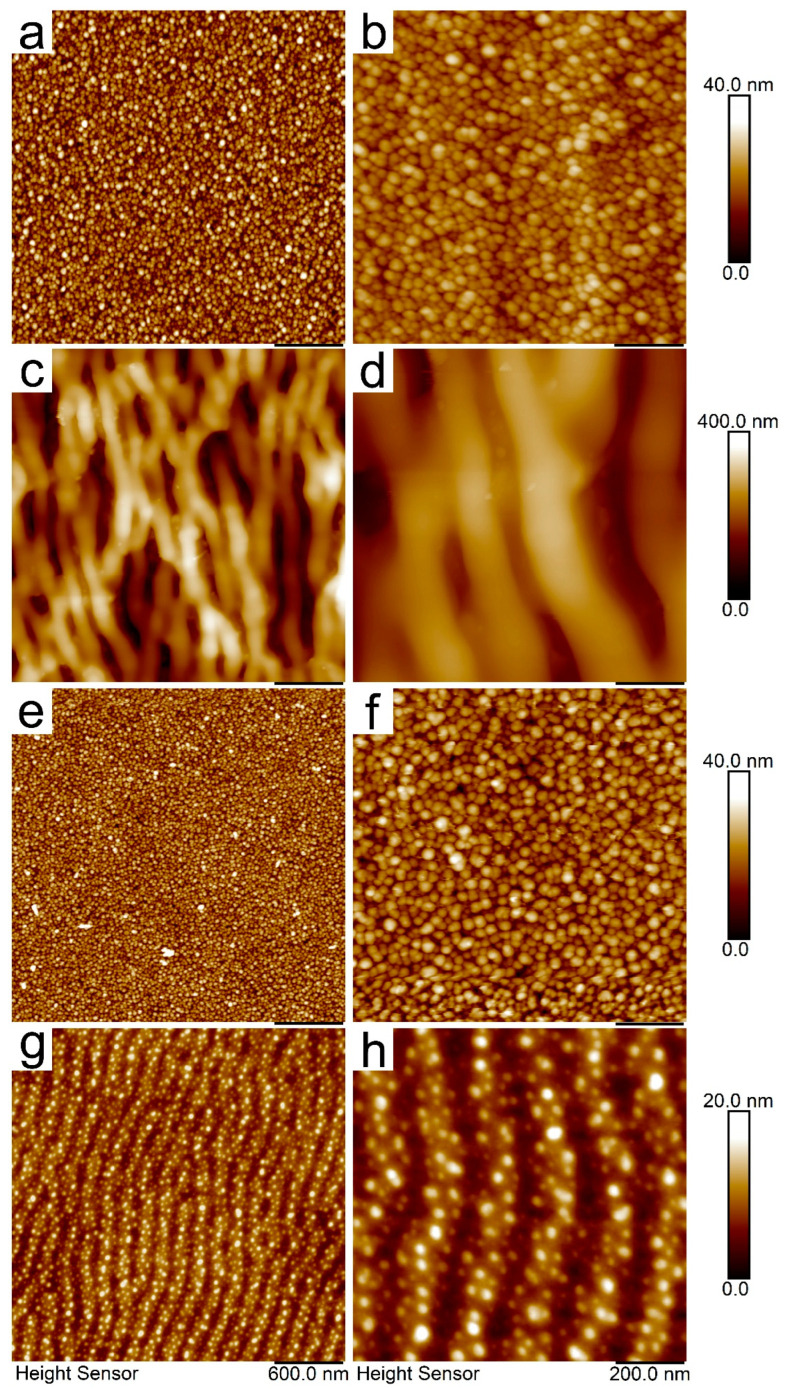
AFM images of AgNP-immobilised PET (**a**,**b**), and AgNP-immobilised PET after applied modifications with KrF laser (**c**,**d**), Ar plasma (**e**,**f**), and GaN laser (**g**,**h**). The images in the right column represent large area view (scale bar 600 nm), images in the left column represent detailed views (scale bar 200 nm).

The surface morphologies of PET decorated with AgNPs (PET/Ag), as well as samples after applied modifications were performed by KrF laser (PET/Ag/k7), GaN laser (PET/Ag/g60), and Ar plasma (PET/Ag/p60), were measured by AFM and are depicted in Figure 2. The *R*_a_ and SAD values related to specific AFM scans (3 μm ^(1)^ and 1 μm ^(2)^) are summarised in Table 1.

Obviously, on the basis of the AFM data presented in Figure 2a,b, we successfully decorated the PET foil with AgNPs. The particles were evenly spread over the polymer surface and their structural parameters (size and shape) remained preserved even after the immobilisation process (see Figure 1). Surface roughness slightly increased compared to the pristine PET, which was typically below 1 nm (see Table 1); however, the original planar character of the foil remained unchanged.

The development of the surface morphology after the applied modifications is shown in Figure 2c–h. More specifically, Figure 2c,d shows the surface morphology of the AgNP-decorated PET after consecutive modification with the KrF laser. The surface morphology changed dramatically, and one can observe the development of a periodically ripped structure. These features are known as laser-induced periodic surface structures (LIPSSs). Typically, in the case of pristine polymers, modification with linearly polarised light from a KrF laser produces much more regular LIPSSs because of the different refractive index [26], which is a crucial parameter in the formation process of these structures. The presence of AgNPs in the thin surface layer of the polymer changed the refractive index, and these changes in the dielectric properties had an obvious impact on the regularity of the LIPSSs. Hand in hand with LIPSS formation, the surface roughness increased significantly, and silver nanoparticles were almost undetectable in the AFM scans. This was most likely due to two possible effects: (i) redistribution of AgNPs during the process of LIPSS formation, which are apparently located in the ridges between the adjacent ripples, and (ii) incorporation of particles into deeper regions of the polymer due to the combination of conversion of radiation into heat and the force effects of polarised light [25] (see Figure 2d).

In contrast, the effect of plasma modification was almost unobservable in the AFM images (Figure 2e,f). The surface structure resembled that of AgNP-decorated PET, which was also supported by the *R*_a_ parameter (Table 1). However, in the FEG-SEM images, one can identify tiny gaps between individual particles (to be discussed later).

Finally, modification with the GaN laser transformed the originally planar polymer surface into a slightly modulated LIPSS-like structure that was much more regular compared to the KrF laser-modified samples (Figure 2g,h). However, it should be noted that the modulation was very flat compared to that observed in Figure 2c,d (see the individual Z-axis scaling). This was due to a different mechanism of LIPSS formation, which in the case of the GaN laser was caused by scanning of the laser beam over the surface of the sample. Owing to light-to-heat conversion in AgNPs, LIPSS formation is governed by the so-called Marangoni effect [27], which consists of polymer mass redistribution governed by the surface tension gradient. In contrast to the LIPSSs formed by KrF irradiation, the AgNPs appeared to be homogeneously distributed over the LIPSSs without spatial preference (ripple ridges and valley). The flat nature of the GaN laser-modified samples was demonstrated by the corresponding *R*_a_ values (see Table 1). It was also obvious that the concentration of AgNPs on the very surface was much lower than that of an unmodified AgNP/PET sample, most likely due to the penetration of AgNPs into the deeper area of the modified polymer.

**Figure 3:**
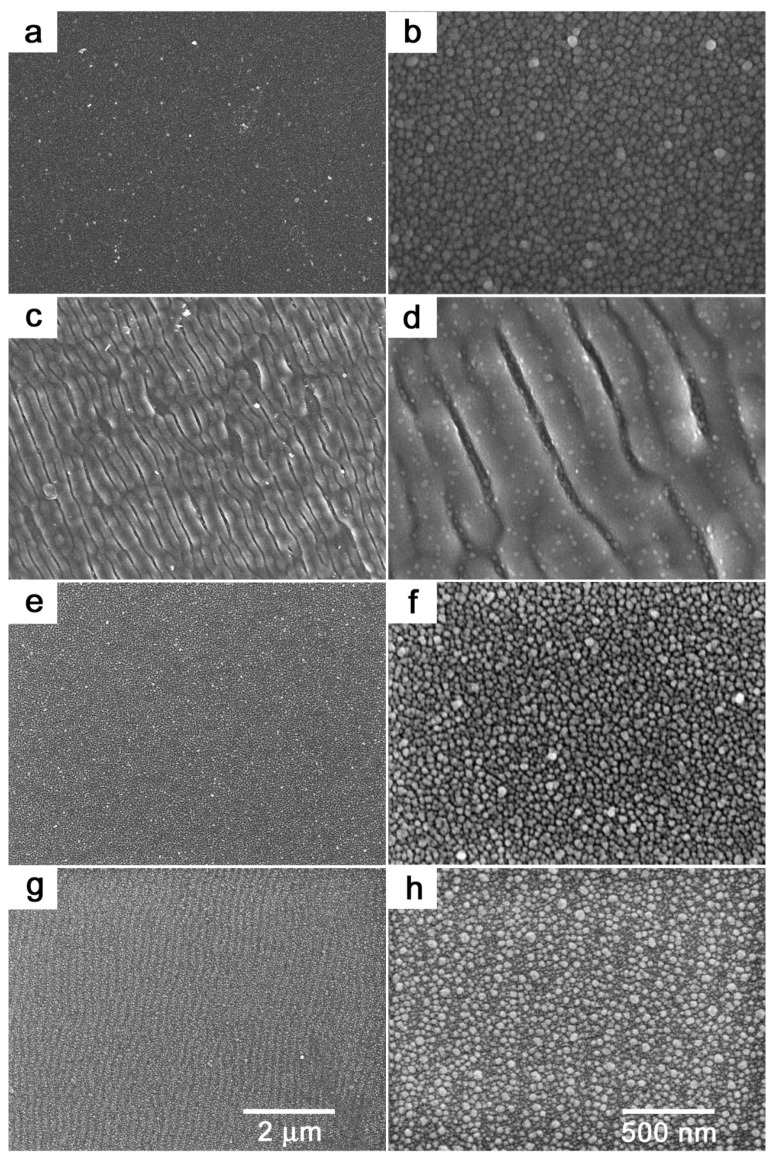
FEG-SEM images of AgNP-immobilised PET (**a**,**b**), and AgNP-immobilised PET after applied modifications with KrF laser (**c**,**d**), Ar plasma (**e**,**f**), and GaN laser (**g**,**h**). The images in the left column represent detailed views.

In order to better visualise AgNPs on the PET surface, we performed supplementary FEG-SEM analysis, which provided a more detailed view of the sample surface because of excellent material contrast between the metal and polymer. The corresponding SEM images are shown in Figure 3. Generally, the SEM measurements confirmed the conclusions based on the AFM analysis. The presence of AgNPs on the PET foil is clearly visible in Figure 3a,b. New details emerged in the case of the KrF laser-irradiated samples, with AgNPs present also on the top of the LIPSS ridges, although in far fewer numbers than in the areas between them (see Figure 3b,c). SEM analysis further revealed a particular change in the surface morphology of the plasma-treated samples, where one could identify tiny gaps between individual particles in the detailed view (Figure 3f). This was most likely due to the effect of Ar plasma on the underlying polymer resulting in scission of the polymeric macromolecules. Finally, SEM micrographs of samples treated with the GaN laser confirmed the findings from the AFM analysis. Due to the thermal effect of light at 405 nm [25], which is pretty close to the absorption maxima of round-shaped silver nanoparticles (~410 nm), light corrugation reminiscent of LIPSS was observed (Figure 3g,h). Moreover, in the detailed view (Figure 3h), it was obvious that individual particles changed their distribution over the polymer surface. The bigger particles seemed to be brighter than the smaller ones, which likely pointed to incorporation of smaller particles into deeper regions of the polymer surface.

Knowledge of the concentration of silver on the surface of the polymer is essential information to demonstrate successful immobilisation of AgNPs. Although the presence of AgNPs was apparent from both AFM and SEM analyses, we performed XPS analysis to obtain quantitative data. Table 2 summarises the concentrations of silver Ag3d, carbon C1s, and oxygen O1s derived from XPS, together with detected impurities. Data on pristine PET are also included and were calculated based on polymer stoichiometry. The concentration of Ag in AgNP-immobilised PET reached a maximum value and gradually decreased with applied treatment. After KrF laser treatment, the silver concentration decreased to 1.6 at.%, which indicated that AgNPs were immersed in deeper regions under the surface of the polymer, as they could not be removed from the sample by modification itself. Together with the decrease in the concentration of Ag, the concentration of O increased at the expense of the concentration of C, which pointed to the incorporation of new oxygen-containing groups into the polymer. Typically, carbonyl, carboxyl, and hydroxyl groups emerge in the polymer chain after laser treatment [28]. A similar effect could also be observed in the case of GaN laser treatment; however, the decrease in Ag concentration was less pronounced. This corresponded well with the SEM analysis (see Figure 3h), where one could identify a slight incorporation of smaller particles into the polymer volume. The silver concentration after plasma modification remained nearly unchanged (see Table 2), which corresponded with both the AFM and SEM analyses, and the plasma-treated surface looked very similar to AgNP-immobilised PET. The detected impurities in the case of the Ar plasma-modified samples most likely originated from the treatment chamber, as they predominately contained iron and aluminium.

**Figure 4:**
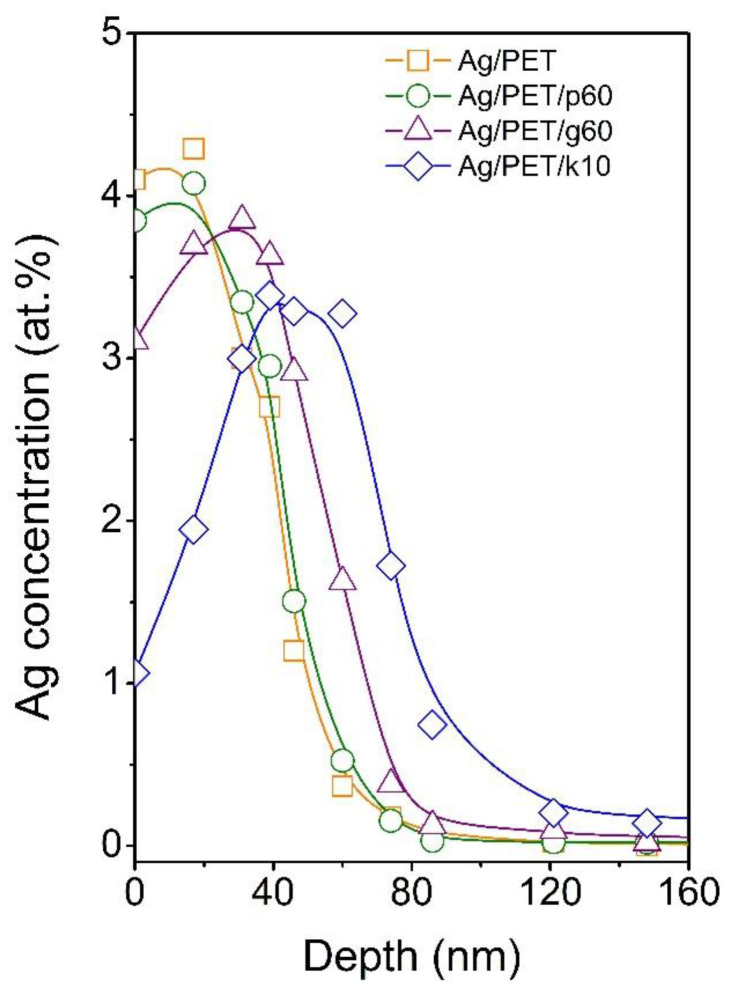
Concentration profiles of Ag in AgNP-immobilised PET (Ag/PET) and AgNP-immobilised PET after applied modifications with Ar plasma (Ag/PET/p60), GaN laser (Ag/PET/g60), and KrF laser (Ag/PET/k10), as measured by RBS.

Data from the elemental analysis of the surface indicated that the applied modifications affected the distribution of AgNPs toward the polymer interior. The possibility of controlling the distribution of nanoparticles in the surface layer of the polymer is absolutely essential with regard to the use of such treated polymers in the preparation of PET membranes with pores containing AgNPs. To reveal the penetration depth of AgNPs beneath the PET surface after applied modification, RBS analysis was performed. The concentration profiles of Ag in AgNP-immobilised PET (Ag/PET) and samples after individual modifications applying plasma treatment (Ag/PET/p60), GaN laser treatment (Ag/PET/g60), and KrF laser treatment (Ag/PET/k10) are shown in Figure 4. It was obvious that the concentration profiles of Ag in immobilised and plasma-treated PET were quite similar, with maxima at the very surface of the polymer. Toward the polymer volume, the Ag concentration gradually decreased, and at a depth of approximately 80 nm, it reached almost zero concentration. The situation differed slightly in the case of GaN laser treatment. The maximum of the concentration profile was shifted below the polymer surface at about 30 nm; however, at the same time, the maximum depth at which silver was still detectable did not change much (compared to the Ag/PET and Ag/PET/p60 samples). The most significant change in the concentration profile of silver occurred after KrF laser treatment. The concentration of Ag at the very surface decreased dramatically as the profile maximum shifted at a depth of approximately 45–50 nm. At the same time, the maximum penetration depth increased to ~120 nm.

**Figure 5:**
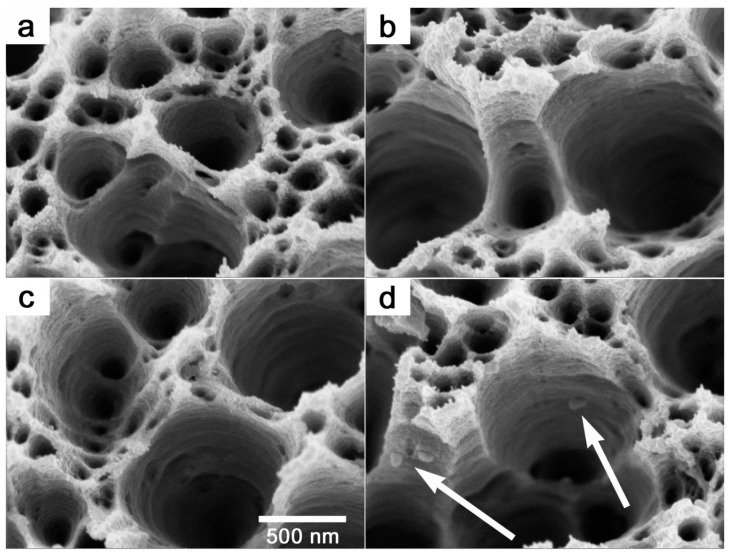
FEG-SEM micrographs of ion-track polymer membranes in AgNP-immobilised PET (**a**) and AgNP-immobilised PET after applied modifications with Ar plasma (**b**), GaN laser (**c**), and KrF laser (**d**). The arrows show anchored silver nanoparticles on the pore walls.

In further experiments, we used PET foils with immobilised AgNPs both unmodified and modified as templates for the preparation of polymeric membranes. These experiments were intended to show that changing the distribution of AgNPs toward the bulk of the polymer can lead to enrichment of the pores of polymer membranes prepared by a combination of ion tracks and chemical etching methods with silver nanoparticles. The SEM micrographs of the prepared membranes are presented in Figure 5. It was obvious that the combination of ion tracks with wet chemical etching led to the creation of pores in PET foil. However, no AgNPs could be detected in the membrane’s pores in the case of immobilised PET, nor in the plasma- or GaN laser-treated samples. This was most likely due to the removal of part of the polymer surface during the nuclear pore etching process, when, due to the shallow depth of distribution of AgNPs, the particles were removed simultaneously with the polymer. In contrast, a relatively promising result was achieved in the case of KrF laser-modified AgNP-decorated PET, where the presence of silver nanoparticles in the membrane pores could be detected (see the structures indicated by arrows in Figure 5d). To achieve more satisfactory results (increased concentration of AgNPs embedded in polymer pores), it will be necessary to optimise the redistribution process of nanoparticles after their immobilisation on the PET surface. However, the study findings suggest that the additional modification of AgNP-decorated PET with KrF laser radiation could be a suitable tool to meet this criterion. By appropriate adjustment of the process parameters, a method using a GaN laser may also become suitable in the future, since it has the potential to shift the maximum of the concentration distribution curve of immobilised silver nanoparticles to deeper regions below the surface of the polymer. The appropriate combination of both methods may also be potentially interesting.

## 3. Materials and Methods

### 3.1. Materials, Apparatus, and Procedures

Silver nanoparticles in the form of colloid solution in distilled water were prepared by an electrochemical process according to the procedure described in [29]. After synthesis, the concentration of AgNPs was determined by AAS (Agilent 280FS AA flame atomiser spectrometer, Agilent Technologies, Santa Clara, CA, USA). For further experiments, the AgNP concentration was set to 30 mg·L^−1^ by diluting with a 1 mM solution of sodium citrate (Na_3_C_6_H_5_O_7_, Sigma-Aldrich Co., St. Louis, MO, USA). Before the immobilisation process, AgNP colloids were characterised by TEM.

AgNP immobilisation process was carried out using a KrF excimer laser (COMPex Pro 50F, Coherent, Inc., Saxonburg, PA, USA, wavelength 248 nm, pulse duration 20–40 ns, repetition rate 10 Hz, 6000 pulses). PET foil (Hostaphan^®^ Mitsubishi Polyester Films, Wiesbaden, Germany, thickness 19 mm, dimensions 45 × 95 mm^2^) was centered in a spectroscopic cuvette (Starna Scientific Ltd., Ilford, UK, type 3/Q/100, volume 35 mL) and charged with 30 mL of AgNP colloid. The cuvette was mounted to a PC-controlled step-motor, allowing automatic movement of the sample relative to the laser beam and preparation of samples with a treated surface area exceeding that defined by the aperture. In this setup, the PET foil was irradiated by 6000 laser pulses through a linear polariser (UV-grade fused silica prism, model PBSO-248-100, Coherent Inc., Saxonburg, PA, USA) and an aperture of 5 × 10 mm. The immobilisation process was carried out with a laser fluence of 7 mJ cm^−2^, so that the planar morphology of the PET foil remained unchanged after the procedure.

Plasma treatment of PET with immobilised AgNPs was performed using a BAL-TEC SCD050 sputter coater (Pfäffikon, Switzerland) in etching mode. The experiment was conducted under the following conditions: pressure 4–6 Pa, gas argon, discharge power 8 W, electrode distance 50 mm, exposure time 60 s (Ag/PET/P60).

Laser modification of PET with immobilised AgNPs was performed using a KrF excimer laser (Coherent, Inc., Saxonburg, PA, USA) and GaN laser (Olympus Corporation, Shinjuku, Japan). Samples were irradiated with a KrF excimer laser on the same device and under the same conditions as in the process of AgNP immobilisation with a laser fluence of 10 mJ∙cm^−2^ (Ag/PET/k10) and with a GaN laser (wavelength 405 nm, objective 50×, zoom 6×), which was a part of the Olympus LEXT OLS3100 confocal laser scanning microscope (Olympus Corporation, Shinjuku, Japan). The exposure time was 60 s (Ag/PET/g60), and the modified area was 30 × 50 µm^2^.

Membrane preparation was carried out by wet chemical etching of ion tracks in AgNP-immobilised/modified PET films previously irradiated by Xe^+26^ ions with an energy of 1.2 MeV/u (ion irradiation of PET films was carried out at JINR Dubna by Dr. P.Y. Apel). An asymmetric etching protocol was applied to prepare membranes on irradiated foils, producing conical-shaped pores with the base orientated towards the etching side. For the etching procedure, 9 M NaOH solution was used at a temperature of 65 °C and etching time of 45 min.

### 3.2. Analytical Methods

Atomic absorption spectroscopy (AAS) was used to determine the concentration of synthesised AgNPs using an Agilent 280FS AA flame atomiser spectrometer (Agilent Technologies, Santa Clara, CA, USA). The measurement error was less than 4%.

UV-Vis absorption measurements were carried out on a Perkin Elmer Lambda 25 UV-Vis spectrophotometer (Waltham, MA, USA, deuterium and halogen lamp light sources, range 350–800 nm, room temperature) equipped with a module for solid sample measurement. The scanning speed was set to 240 nm min^−1^ with a data collection interval of 1 nm.

Transmission electron microscopy (TEM) characterisation of the AgNP colloid was performed using a JEOL JEM-1010 instrument (JEOL Ltd., Akishima, Tokyo, Japan) operated at 80 kV. Particle size was measured from the TEM micrographs and calculated with respect to at least 500 particles, using AnalysSIS 2.0 software (JEOL Ltd., Akishima, Tokyo, Japan). A drop of colloidal solution was placed on a copper grid coated with thin amorphous carbon film on filter paper. The samples were air-dried and kept under vacuum in a desiccator before being placed on a specimen holder.

Atomic force microscopy (AFM) analysis was performed on a Dimension ICON instrument (Bruker Corp., Billerica, MA, USA) in ScanAsyst^®^ tapping mode in air, using an antimony-doped silicon probe type RTESPA-150 (Bruker Corp., USA) with an elasticity constant of 0.4 N·m^−1^ and natural frequency of 70 kHz. Data were acquired at a scan rate of 0.5 Hz and evaluated using NanoScope^®^ Analysis software 2.0. The surface characteristics of the scanned area such, as the surface roughness (*R*_a_) and surface area difference (SAD), were derived from AFM analysis. The *R*_a_ and SAD values are defined as the absolute average relative to the central plane of the sample and the ratio of the difference between the measured area and the scanned area to the scanned area, respectively.

Field emission gun scanning electron microscopy (FEG-SEM) was used for surface visualisation of the AgNP-immobilised/modified PET foil. Measurements were carried out using the MAIA3 high-resolution FEGSEM microscope (Tescan, Brno, Czech Republic) equipped with detectors for secondary and backscattered electrons, operating in high-resolution mode at an accelerating voltage of 3 kV.

The chemical composition of the surface layer was determined by X-ray photoelectron spectroscopy (XPS) using an Omicron Nanotechology ESCAProbeP spectrometer (Omicron Nanotechnology Ltd., East Grinstead, UK). The X-ray source was monochromated at 1486.7 eV with a step size of 0.05 eV. Spectrum evaluation was carried out by CasaXPS software 3.0. The uncertainty of the measurement was less than 3%.

The penetration of AgNPs into the polymer surface was analysed by Rutherford backscattering spectrometry (RBS). RBS spectra were measured on a 3 MV Tandetron MC 4130 accelerator (High Voltage Engineering Europa BV, Amersfoort, The Netherlands) using 2.0 MeV ^4^He^+^ ions at a scattering angle of 170° in the Cornell geometry. The Ultra-Ortec PIPS detector (Oak Ridge, TN, USA) solid angle was 2.612 mSr, the spectrometer energy resolution for 2.0 MeV ^4^He^+^ ions was defined at full width at half maximum (FWHM) = 12 keV, and the beam spot area on the sample was 1 × 1 mm^2^. The typical beam current was 5 nA.

## 4. Conclusions

In this study, we aimed to enrich the walls of etched ion-track PET membrane pores with silver nanoparticles. To achieve this, we introduced a novel approach of applying a treatment procedure to AgNP-immobilised PET, the essence of which lay in redistribution of nanoparticles from the very surface to the deeper regions of the polymer. The redistribution of the AgNP profile was crucial because, during the etching procedure of ion-track PET with immobilised AgNPs, the top polymer layer was removed. We demonstrated that except for plasma treatment, both laser irradiation methods (KFr and GaN) were promising in this regard; however, it is necessary to focus on the optimisation of both processes in the future, especially in terms of applied fluencies and treatment times to achieve more promising results. We believe that our technology may facilitate subsequent grafting of pores with stimuli-responsive molecules and will be adapted in the future for the construction of intelligent membranes with controllable transport properties.

## Figures and Tables

**Table 1:** Average roughness (*R*_a_) and surface area difference (SAD) derived from AFM analysis for large (1) and small (2) area scans presented in Figure 2.

Polymer/ Modification	Parameters Derived from AFM Analysis			
	*R*_a_ ^(1)^ (nm)	*R*_a_ ^(2)^ (nm)	SAD ^(1)^ (%)	SAD ^(2)^ (%)
PET	0.9	1.1	1.1	1.2
PET/Ag	3.8	4.2	17.4	16.8
PET/Ag/k7	51.2	48.8	39.7	32.7
PET/Ag/p60	3.7	4.4	14.6	15.1
PET/Ag/g60	2.1	2.9	4.6	5.8

**Table 2:** Concentrations of silver Ag3d, carbon C1s, oxygen O1s, and impurities (in at. %) in pristine (PET), AgNP-immobilised (PET/AgNPs), and AgNP-immobilised PET after applied modifications, derived from XPS analysis. Modifications were conducted by KrF laser (PET/AgNPs/k7), Ar plasma (PET/AgNPs/p60), and solid-state GaN laser (PET/AgNPs/g60).

Polymer/ Modification	Element Concentration (at. %)			
	Ag	C	O	Impurities
PET	-	76.6	23.4	-
PET/Ag	23.7	59.1	17.2	-
PET/Ag/k7	1.6	69.7	28.7	-
PET/Ag/p60	20.8	54.1	22.9	2.2
PET/Ag/g60	13.6	64.6	21.8	-

## Data Availability

The data presented in this study are available upon request from the corresponding author.
